# Evaluation of Diagnostic Recommendations Embedded in Medication Alerts: Prospective Single-Arm Interventional Study

**DOI:** 10.2196/70731

**Published:** 2025-05-27

**Authors:** Yu-Chen Liu, Guan-Ling Lin, Jeremiah Scholl, Yi-Chun Hung, Yu-Jing Lin, Yu-Chuan Li, Hsuan-Chia Yang

**Affiliations:** 1 School of Nursing College of Medicine National Taiwan University Taipei Taiwan; 2 Graduate Institute of Biomedical Informatics College of Medical Science and Technology Taipei Medical University Taipei Taiwan; 3 AESOP Technology Taipei Taiwan; 4 Department of Pharmacy Tungs' Taichung MetroHarbor Hospital Taichung Taiwan; 5 International Center for Health Information Technology (ICHIT) Taipei Medical University Taipei Taiwan; 6 Clinical Big Data Research Center Taipei Medical University Hospital Taipei Taiwan; 7 Research Center of Big Data and Meta-analysis Wanfang Hospital Taipei Medical University Taipei Taiwan

**Keywords:** clinical decision support system, potentially inappropriate prescribing, diagnostic recommendations, outpatient care, machine learning, alert fatigue, MedGuard

## Abstract

**Background:**

Potentially inappropriate prescribing in outpatient care contributes to adverse outcomes and health care inefficiencies. Clinical decision support systems (CDSS) offer promising solutions, but their effectiveness is often constrained by incomplete medical records.

**Objective:**

This study aims to evaluate the effectiveness of a machine learning–based CDSS for enhancing diagnostic recommendations, which are system-suggested diagnoses, ensuring that each prescribed medication has a corresponding diagnosis documented and meets medication appropriateness.

**Methods:**

This prospective single-arm interventional study was conducted over 1 year in the outpatient departments of a hospital. The system provided diagnostic recommendations based on machine learning algorithms trained on data from the National Health Insurance Research Database. Outcome measures included alert rates, acceptance rates of diagnostic recommendations, and variability in system performance across specialties. Descriptive and trend analyses were used to evaluate the system’s effectiveness.

**Results:**

This study included 438,558 prescriptions from 44 physicians across 23 specialties, involving 125,000 unique patients in the outpatient departments of a regional teaching hospital. MedGuard, embedded with diagnostic recommendations, achieved an overall alert rate of 2.28% and a diagnostic recommendation acceptance rate of 56.55%. All accepted recommendations resulted in actionable changes, including prescription adjustments or the addition of missing diagnoses. Ophthalmology achieved the highest acceptance rate at 96.59%, while rheumatology, surgery, psychiatry, and infectious disease recorded acceptance rates of 0%, 0%, 24.74%, and 35%, respectively. Over the years, acceptance rates for potentially inappropriate prescriptions stabilized at 51%, despite increasing prescription volumes.

**Conclusions:**

This study demonstrates the potential of embedding diagnostic recommendations into alerts within a machine learning–based clinical decision support system to improve diagnostic completeness and support safer outpatient care. Future efforts should refine alerts to align with specialty-specific workflows and validate their effectiveness in diverse clinical settings.

## Introduction

Ensuring prescription accuracy and medication appropriateness is fundamental to patient safety and effective health care delivery [1,2]. However, potentially inappropriate prescribing (PIP)—including overprescribing, underprescribing, and prescribing without appropriate indication—remains prevalent, particularly in outpatient settings and among older adults with polypharmacy [3-5]. Inappropriate prescriptions can lead to adverse drug events, hospitalizations, and increased health care costs. For example, a national study in the United States found that from 2014 to 2018, 43 billion doses of potentially inappropriate medications (PIMs) were dispensed to Medicare beneficiaries, costing US $25.2 billion [6].

Clinical decision support systems (CDSS), typically integrated with computerized physician order entry (CPOE) systems, are predominantly rule-based, limiting their ability to capture complex disease-medication (DM) relationships and off-label use [7-9]. These alerts frequently lack relevance, contributing to alert fatigue and low acceptance rates [10-12]. For example, Zwietering et al [13] found that while a rule-based CDSS generated an average of 4.8 remarks per patient, 85% of the CDSS remarks required no action. A systematic review has revealed alert override rates of 46.2% to 96.2%, with the appropriateness of these overrides varying between 29.4% and 100% [11]. Drug-disease interaction and age-based alerts were frequently overridden due to insufficient clinical context [12].

Incomplete diagnostic documentation in electronic health records is a key contributor to medication without indication, a frequent form of PIP that increases the risk of adverse drug events and inappropriate prescribing [13,14]. Missing diagnoses can lead to clinically appropriate medications being flagged as inappropriate, limiting the utility of CDSS alerts. Studies have shown that automated tools can enhance diagnostic completeness, improve the accuracy of electronic health record data, and support safer prescribing [14-16].

Machine learning–based systems offer new opportunities to overcome the limitations of traditional CDSS [17-20]. Leveraging machine learning, MedGuard (developed by AESOP Technology, Taipei, Taiwan) was developed to identify medications without matching diagnoses [21,22]. A proposed solution further involves using large-scale prescription data and advanced analytics to provide evidence-based diagnostic recommendations at the point of care [21], thereby enhancing diagnostic completeness, improving alert relevance, and reducing alert fatigue. Thus, this study aims to evaluate the effectiveness of a machine learning–based CDSS for enhancing diagnostic recommendations and medication appropriateness when deployed in the outpatient setting at a regional teaching hospital in Taiwan.

## Methods

### Study Setting

This clinical trial was conducted in the outpatient departments of Tungs’ Taichung MetroHarbor Hospital, a regional teaching hospital in Taichung, Taiwan. The hospital has 1160 beds, spans 38 specialties, and employs over 2000 staff members. It serves as a regional hub for advanced medical care and education. All data were anonymized before analysis to protect patient privacy and confidentiality. This study adheres to the Transparent Reporting of Evaluations with Nonrandomized Designs (TREND) guidelines for reporting nonrandomized evaluations of behavioral and public health interventions (Multimedia Appendix 1) [23].

### Study Design

The study spanned from January to December 2023, collecting medical records and prescription behaviors from 44 physicians across 23 departments. MedGuard was initially introduced in January 2023 across 14 departments, with additional departments onboarded in March (family medicine, nephrology, and surgery), July (dermatology, orthopedics, others, and plastic surgery), August (pediatrics), and September (emergency medicine; Table S1 in Multimedia Appendix 2). Although the study covered a wide range of departments, the total number of physicians was 44 because some physicians provided cross-departmental outpatient consultations. For example, surgeons practiced in both general surgery and neurosurgery, family medicine physicians also covered surgery, and neurologists served in both neurology and neurosurgery departments.

The 1-year study period was selected as it sufficiently captures adoption patterns and system performance, supported by previous observations of unmodified MedGuard system versions, which were typically observed for one month to one year [21,22]. Additionally, approximately half of the rule-based CDSS studies were observed within one year [11]. During the initial implementation phase of MedGuard, participation was limited, with only 17 physicians using the system in the early stage. Over time, the adoption rate gradually increased, reaching 39 physicians by the seventh month. By the end of December, a total of 44 physicians had used MedGuard.

**Figure 1:**
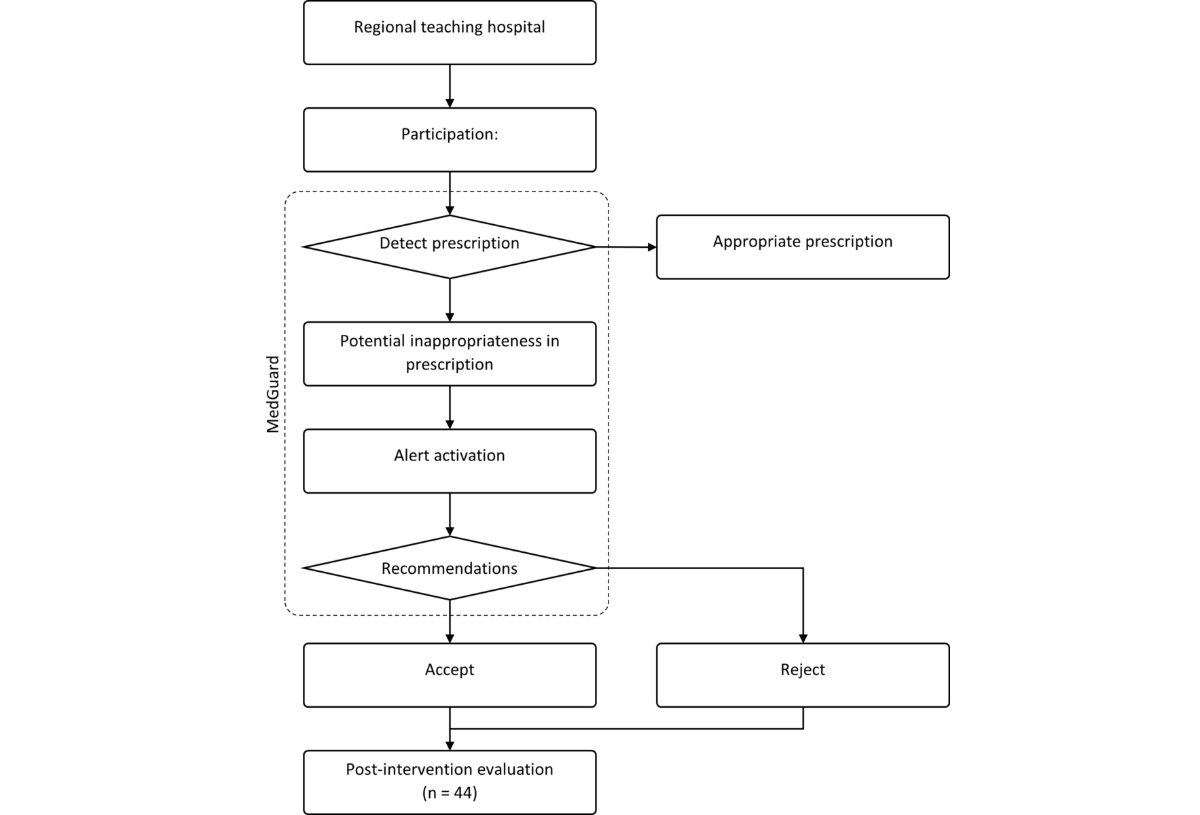
Study workflow.

The clinical workflow is illustrated in Figure 1. The hospital uses a homegrown CPOE system specifically designed for outpatient physicians to manage all diagnostics, medications, and treatment orders. The CDSS, MedGuard, was integrated with the existing CPOE system. AESOP Technology developed both CDSS systems.

### Prescription Appropriateness Detection

**Figure 2:**
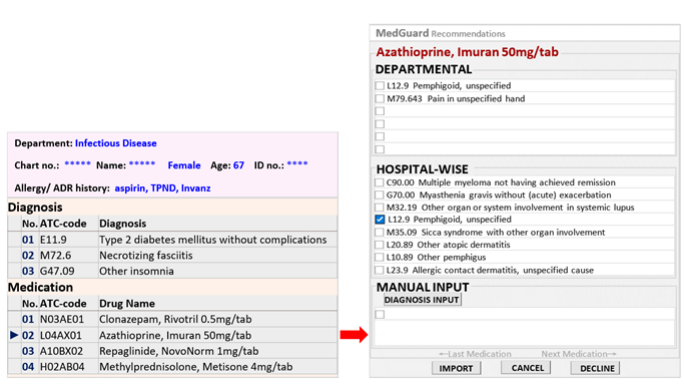
Clinical decision support system: MedGuard.

MedGuard detects prescription appropriateness by alerting physicians when PIMs are prescribed. Its algorithm is based on data mining techniques applied to Taiwan’s National Health Insurance Research Database, encompassing 23 million individuals over 5 years, totaling 1.3 billion prescriptions. It identifies associations between DM and medication-medication pairs using q-values to rank diagnostic relevance [24,25]. The model was validated across 5 Taiwanese hospitals, consistently demonstrating high accuracy (over 80%) and sensitivity ranging from 80% to 96%, ensuring reliable diagnostic suggestions in clinical practice. MedGuard assesses whether the prescribed drugs are suitable when a physician issues a prescription using a large table of white-listed DM and medication-medication pairs constructed from these associations. The term “prescription” refers to a single prescription sheet (or prescription order) that may include multiple diagnoses and medications prescribed to a patient at one time. PIM is defined as a medication that is prescribed without appropriate diagnostic justification, as determined by MedGuard in this study. If the physician attempts to prescribe a medication, but the diagnosis or other information in the patient chart or prescription medications cannot justify the medication, it is flagged as a PIM [21]. For instance, as shown in the prescription sheet in Figure 2, azathioprine, an immunosuppressant, would be flagged as a PIM when prescribed for conditions such as type 2 diabetes mellitus, necrotizing fasciitis, or insomnia, due to its low association with these diagnoses. Consequently, the prescription would also be considered a PIP in this study because it contains at least one PIM.

### Diagnostic Recommendations Embedded in Alerts

In previous studies [21], it was revealed that most PIMs detected by models were related to diagnostic omissions, including missed diagnoses of chronic disease prescriptions or symptomatic diagnoses such as inflammation, fever, pain, and insomnia. Our study enhanced the MedGuard system [26] by embedding diagnostic recommendations into alerts for PIMs, suggesting suitable diagnoses for medications that lack corresponding diagnoses, based on age- and gender-specific correlations between medications and diagnoses. In this study, diagnostic recommendations refer specifically to system-suggested diagnoses intended to ensure that each prescribed medication has a corresponding diagnosis documented in the patient’s chart. As illustrated in the right part of Figure 2, these recommendations are based on real-world DM associations rather than drug labels. The recommendations are tailored to medical specialties, age groups, and gender, ensuring higher clinical relevance. If physicians find the suggested diagnoses unsuitable, they can manually input diagnosis codes with one click, enabling future personalized medication recommendations.

### Data Collection

The study collected the following data: (1) physician prescription data, such as deidentified physician ID, medical specialty, and diagnosis (the *ICD-10-CM* [*International Classification of Diseases, Tenth Revision, Clinical Modification*]) [27]; (2) medication information, including National Health Insurance code [28], anatomical therapeutic chemical (ATC) code [29], frequency, dosage, and duration of treatment; (3) patient data, including deidentified patient ID, age, and gender; and (4) recommendation data, including PIMs, diagnostic recommendations, and physician acceptance or rejection of these recommendations.

### Outcomes Measures and Statistical Analysis

The frequency of PIM alerts, diagnostic recommendations provided, and the acceptance rate of these recommendations were measured. The acceptance rate was defined based on whether physicians selected and imported the suggested diagnosis into the patient's diagnostic data or rejected it by clicking “Cancel” or “Decline.” If any recommended diagnoses within a PIP were accepted and adjusted—either by directly adopting the suggested changes or through manual modifications—it was considered accepted, otherwise, it was considered rejected. Overall, 82.52% of PIPs had only one PIM, 13.67% had two, 3% had three, and 0.81% had four to six, with an average of 1.22 PIMs per PIP.

To illustrate how the system operates in different clinical scenarios, we selected 4 representative prescribing scenarios for detailed analysis. These scenarios covered commonly prescribed medications (amlodipine), drugs with multiple specialty-specific indications (nifedipine), drugs with multiple therapeutic indications (propranolol), and high-risk medications requiring careful diagnostic consideration (methotrexate). Descriptive statistics were used to characterize prescriptions, recommendations, and acceptance, with PIP and PIM prevalence expressed as percentages. PIM diagnostic recommendation acceptance rates were also calculated. Rates and acceptance rates with 95% CIs were estimated using the Wilson score interval. Logistic regression models were applied to evaluate trends over time and differences across departments and ATC categories. All models were adjusted for patient age and sex. All analyses were performed using SAS version 9.4 (SAS Institute Inc), with statistical significance set at *P*<.05.

### Ethical Considerations

The study was approved by the institutional review board of Tungs’ Taichung MetroHarbor Hospital (approval number 113051). As the research involved the analysis of nonidentifiable prescribing and system log data collected during routine outpatient care, it was exempt from full ethical review under institutional regulations.

## Results

Over 1 year (January 1, 2023, to December 31, 2023), 44 physicians from 23 specialties participated in this study. A total of 438,558 prescriptions were issued, containing 1,242,681 medication items. The AESOP system, MedGuard, reviewed the appropriateness of these prescriptions and generated alerts when PIPs were identified. These alerts included embedded diagnostic recommendations for potentially missing diagnoses. The system detected 10,006 PIPs, resulting in an alert rate of 2.28%. Of these, 5658 prescriptions incorporated at least one embedded diagnostic recommendation, yielding a mean PIP acceptance rate of 56.55%. Within these PIPs, the system generated 12,237 individual diagnostic recommendations based on the medications flagged by the alerts. Physicians accepted 5681 of these recommendations, resulting in a mean PIM diagnostic recommendation acceptance rate of 46.42% (Table S2 in Multimedia Appendix 3).

**Figure 3:**
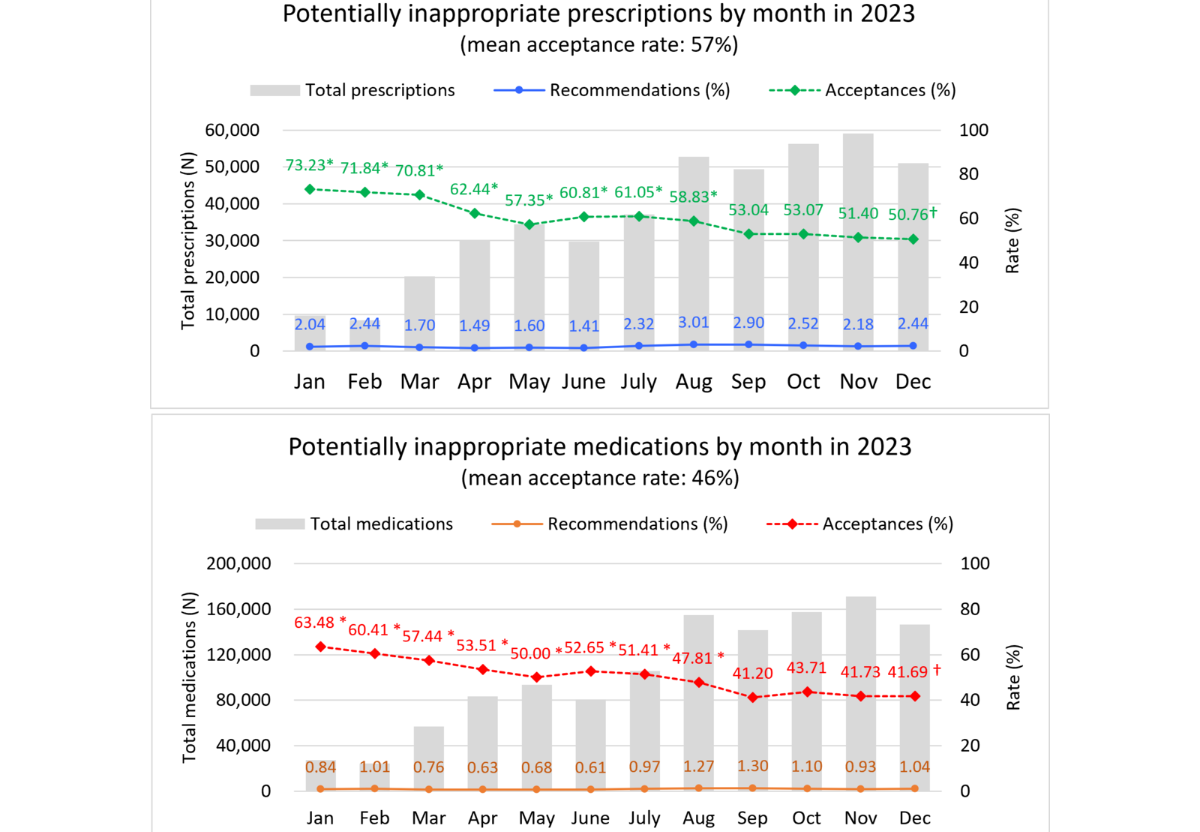
Analysis of alerts and embedded diagnostic recommendations for potentially inappropriate prescriptions across 2023. *Statistically significant difference in the odds ratio of recommendation acceptance (Y=accepted vs not accepted) compared with December, after controlling for age and sex in the logistic regression analysis. †December serves as the reference month in the logistic regression analysis.

Observing the trend, PIP acceptance rates declined from 72% to 51% but stabilized at 51% later in the year, despite rising prescription volumes. Similarly, PIM diagnostic recommendation acceptance rates dropped from 63% to 42% but plateaued at 42%. The proportion of diagnostic recommendations for PIPs (1.41%-3.01%) and PIMs (0.61%-1.27%) remained low throughout the year (Figure 3 and Table S3 in Multimedia Appendix 4). Logistic regression analysis further supported these trends, showing significantly higher acceptance rates from January to August compared with December, followed by a gradual decline and stabilization from September onwards.

**Figure 4:**
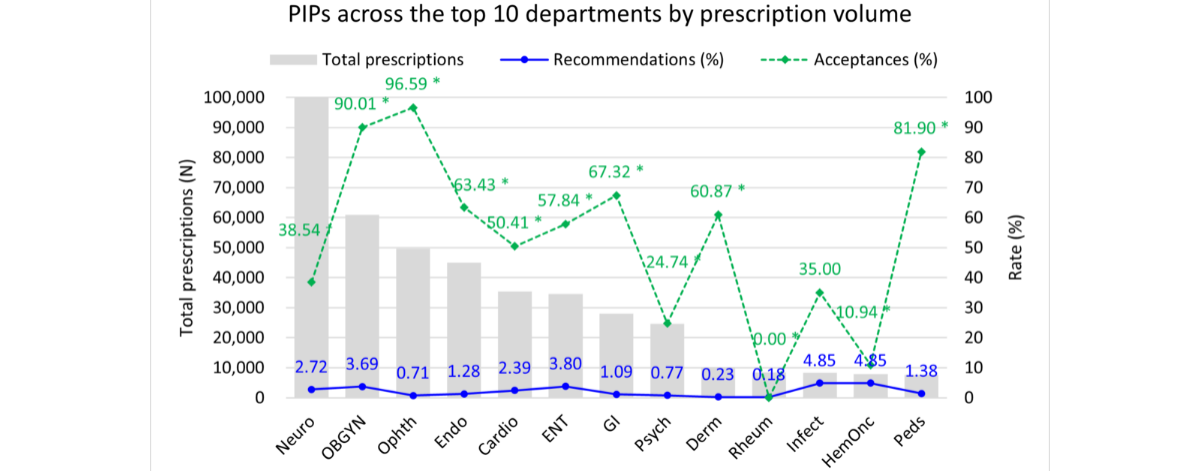
Analysis of alerts and acceptance rate of embedded diagnostic recommendations by department. Cardio: cardiology; Derm: dermatology; Endo: endocrinology; ENT: otorhinolaryngology; GI: gastroenterology; HemOnc: hematology and oncology; Infect: infectious disease; Neuro: neurology; OBGYN: obstetrics and gynecology; Ophth: ophthalmology; Peds: pediatrics; PIP: potentially inappropriate prescriptions; Psych: psychiatry; Rheum: rheumatology. *Statistically significant difference in the odds ratio of recommendation acceptance (Y=accepted vs not accepted) compared with neurology, after controlling for age and sex in the logistic regression analysis. †Neurology serves as the reference department in the logistic regression analysis.

The rates of diagnostic recommendations and their acceptance varied across specialties (Figure 4 and Table S4 in Multimedia Appendix 5). Ophthalmology had the highest acceptance rate (96.59%) with a low recommendation rate (0.71%), followed by obstetrics and gynecology (90.01%) and pediatrics (81.90%). Rheumatology had no accepted recommendations (0%) and the lowest recommendation rate (0.18%). Hematology and oncology also had low acceptance (10.94%) with a recommendation rate of 4.85%. Logistic regression analysis (Table S5 in Multimedia Appendix 6) supported these findings.

**Figure 5:**
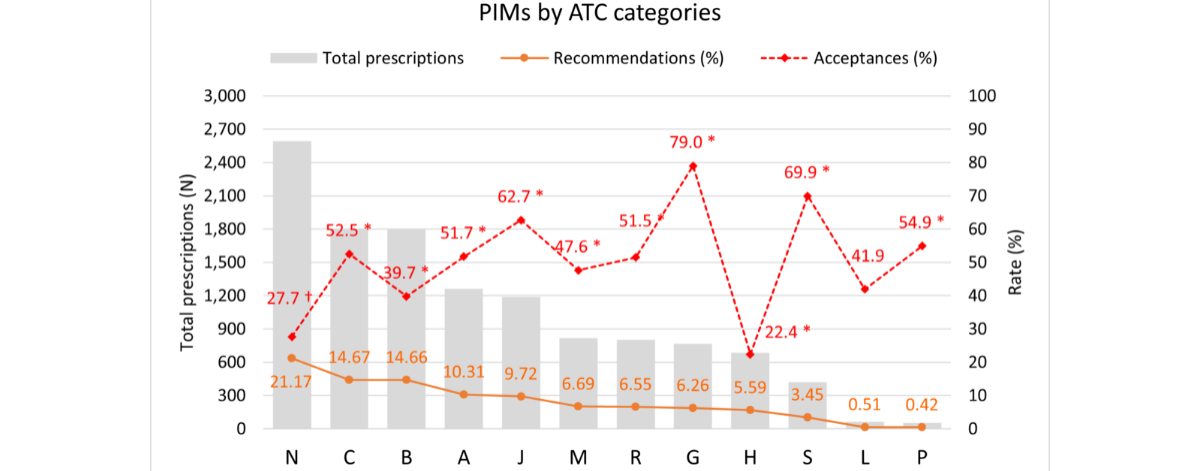
Analysis of alerts and acceptance rate of embedded diagnostic recommendations by anatomical therapeutic chemical categories. A: alimentary tract and metabolism; ATC: Anatomical Therapeutic Chemical Classification System; B: blood and blood forming organs; C: cardiovascular system; G: genitourinary system and sex hormones; H: systemic hormonal preparations (excluding sex hormones and insulins); J: anti-infectives for systemic use; L: antineoplastic and immunomodulating agents; M: musculoskeletal system; N: nervous system; P: antiparasitic products, insecticides, and repellents; PIMs: potentially inappropriate medications; R: respiratory system; S: sensory organs. *Statistically significant difference in the odds ratio of recommendation acceptance (Y=accepted vs not accepted) compared to category N, after controlling for age and sex in the logistic regression analysis. †Category N serves as the reference category in the logistic regression analysis.

The rates of diagnostic recommendations and their acceptance varied across ATC categories (Figure 5 and Table S6 in Multimedia Appendix 7). Among all categories, nervous system drugs (N) accounted for the highest number of PIMs, but the acceptance rate was only 27.7%. In contrast, the genitourinary system and sex hormones (G) showed the highest acceptance rate at 79%, followed by sensory organs (S) at 69.9%, and antineoplastic and immunomodulating agents (L) at 54.9%. Hormonal preparations (H) had the lowest acceptance rate at 22.4% despite a moderate volume of recommendations. Logistic regression analysis supported these findings with the exception that antineoplastic and immunomodulating agents (L) showed no significant difference compared with nervous system drugs (N).

The top 3 most frequently flagged PIPs were acetylsalicylic acid (B01AC06, 568 recommendations in alerts, 171 acceptances), chlorzoxazone (M03BB03, 452 recommendations, 257 acceptances), and paracetamol combinations (N02BE51, 402 recommendations, 184 acceptances; Table 1). By ATC classification, the top flagged categories were nervous system (2591 recommendations, 717 acceptances), cardiovascular system (1795 recommendations, 943 acceptances), and blood and blood forming organs (1794 recommendations, 713 acceptances; Table S7 in Multimedia Appendix 8).

**Table 1:** Top 10 potentially inappropriate medications with embedded diagnostic recommendations and acceptances.

Rank	ATC^a^ code	Drug name	Diagnostic recommendation (alert), n (%)		Acceptance, n (%)	
1	B01AC06	Acetylsalicylic acid	568 (4.64)		171 (30.11)	
2	M03BB03	Chlorzoxazone	452 (3.69)		257 (56.86)	
3	N02BE51	Paracetamol, combinations excluding psycholeptics	402 (3.29)		184 (45.77)	
4	B06AA11	Bromelain	353 (2.88)		162 (45.89)	
5	R06AE09	Levocetirizine	303 (2.48)		162 (53.47)	
6	B03BA05	Mecobalamin	291 (2.38)		105 (36.08)	
7	H02AB02	Dexamethasone	291 (2.38)		34 (11.68)	
8	J01DB01	Cephalexin	277 (2.26)		161 (58.12)	
9	H02AB06	Prednisolone	268 (2.19)		87 (32.46)	
10	J01CR02	Amoxicillin and beta-lactamase inhibitor	249 (2.03)		189 (75.90)	
Sum of the top 10, N (%)				3454 (28.23)		1512 (43.78)

^a^ATC: anatomical therapeutic chemical.

The top three most frequently accepted embedded diagnostic recommendations were essential (primary) hypertension (I10) with 334 acceptances (5.88% of total acceptances), allergic rhinitis, unspecified (J30.9) with 209 acceptances (3.68%), and acute vaginitis (N76.0) with 193 acceptances (3.40%; Table 2).

**Table 2:** Top 10 adjusted diagnoses for potentially inappropriate medications.

Rank	*ICD-10-CM*^a^ code	Disease name	Acceptances, n (%)	
1	I10	Essential (primary) hypertension	334 (5.88)	
2	J30.9	Allergic rhinitis, unspecified	209 (3.68)	
3	N76.0	Acute vaginitis	193 (3.4)	
4	N39.0	Urinary tract infection, site not specified	190 (3.34)	
5	H04.129	Dry eye syndrome of unspecified lacrimal gland	155 (2.73)	
6	E78.5	Hyperlipidemia, unspecified	150 (2.64)	
7	R35.0	Frequency of micturition	139 (2.45)	
8	N30.00	Acute cystitis without hematuria	132 (2.32)	
9	R42	Dizziness and giddiness	130 (2.29)	
10	J03.80	Acute tonsillitis due to other specified organisms	126 (2.22)	
Sum of the top 10, N (%)				1758 (30.95)
Total				5681 (100)

^a^*ICD-10-CM: International Classification of Diseases, Tenth Revision, Clinical Modification*.

We evaluated MedGuard’s adaptability using four representative examples, such as amlodipine, nifedipine, propranolol, and methotrexate, to demonstrate its performance in various prescribing scenarios (Table 3). Among these examples, Amlodipine (C08CA01) generated 146 alerts with an acceptance rate of 63.01%. For nifedipine (C08CA05), a multiuse medication, 37 alerts were triggered, achieving an acceptance rate of 56.76%. Propranolol (C07AA05), a nonselective beta-blocker, generated 172 alerts with an acceptance rate of 48.83%. Finally, Methotrexate (L01BA01), a high-risk immunomodulator, had 8 alerts with a notably high acceptance rate of 87.50%.

**Table 3:** Embedded diagnostic recommendations and acceptances for medications with multiple therapeutic uses.

Scenario	Drug name (ATC^a^ code)	Diagnostic recommendation (alerts)	Acceptances, n (%)	Diagnosis (*ICD*^b^ code): hierarchical acceptance, n (%)
Cardiovascular medication (hypertension)	Amlodipine (C08CA01)	146	92 (63.01)	Essential (primary) hypertension (I10): 92 (100)
Multiuse medication with specialty-specific indications	Nifedipine (C08CA05)	37	21 (56.76)	Preterm labor without delivery, second trimester (O60.02): 15 (71.43) Essential (primary) hypertension (I10): 2 (9.52) False labor before 37 completed weeks of gestation, second trimester (O47.02): 1 (4.76) Preterm labor without delivery, unspecified trimester (O60.00): 1 (4.76) Other specified diseases and conditions complicating pregnancy, childbirth, and the puerperium (O99.89): 1 (4.76) Pregnant state, incidental (Z33.1): 1 (4.76)
Medication with multiple therapeutic indications	Propranolol (C07AA05)	172	84 (48.83)	Palpitations (R00.2): 64 (76.19) Cardiac arrhythmia, unspecified (I49.9): 6 (7.14) Essential tremor (G25.0): 5 (5.95) Anxiety disorder, unspecified (F41.9): 3 (3.57) Hemangioma unspecified site (D18.00): 1 (1.19) Generalized anxiety disorder (F41.1): 1 (1.19) Chronic migraine without aura, not intractable, without status migrainosus (G43.709): 1 (1.19) Migraine, unspecified, not intractable, without status migrainosus (G43.909): 1 (1.19) Tension-type headache, unspecified, not intractable (G44.209): 1 (1.19) Tremor, unspecified (R25.1): 1 (1.19)
High-risk immune-modulator	Methotrexate (L01BA01)	8	7 (87.50)	Atopic dermatitis, unspecified (L20.9): 7 (100)

^a^ATC: anatomical therapeutic chemical.

^b^*ICD: International Classification of Diseases*.

## Discussion

### Principal Findings

This study demonstrates that a machine learning–based CDSS (ie, MedGuard), embedding diagnostic recommendations into medication alerts, can be effectively implemented in clinical practice. It achieved a low alert rate of 2.28% across 438,596 prescriptions and a high acceptance rate of 56.55%. All accepted recommendations resulted in actionable changes, including the addition of missing diagnoses or the adjustment of inappropriate prescriptions. Compared with rule-based CDSS, which have reported override rates ranging from 46.2% to 96.2% [11], machine learning models can better capture specialty-specific prescribing patterns and reduce clinically irrelevant alerts.

When comparing our findings to another machine learning–based CDSS, MedAware (developed by MedAware Ltd) [20] and to previous versions of MedGuard, notable differences were observed in alert rates and acceptance rates. MedAware, implemented in an inpatient internal medicine ward, focused on detecting prescription errors and adverse drug events and reported an alert rate of 0.4%, with 43% of alerts accepted and resulting in prescription modifications. Its alerts covered time-dependent irregularities, dosage outliers, drug overlap, and diagnosis-medication mismatches [20].

In contrast, studies evaluating MedGuard in outpatient care reported higher alert rates and varying acceptance rates when compared with MedAware. Poly et al [22] reported an alert rate of 2.36% and an acceptance rate of 61.30% during a one-month study. However, their definition of acceptance was relatively broad, including cases where alerts were merely acknowledged without actual prescription changes, which may overestimate the true acceptance rate. Chen et al [21] reported a higher alert rate of 3.12% and a lower acceptance rate of 48.88% over 2 years, with only 28.08% of accepted alerts leading to actual prescription changes. This lower modification rate may reflect the broader definition of acceptance used in their study, which included mere acknowledgment of alerts, as well as the absence of embedded diagnostic recommendations that directly address diagnostic omissions.

This study further refined MedGuard’s performance, achieving a lower alert rate and a higher acceptance rate. This improvement highlights the benefits of MedGuard, which not only enhances the clinical relevance of alerts but also helps physicians immediately identify and address diagnostic gaps, enabling safer and more appropriate prescribing. Furthermore, MedGuard’s application across 23 specialties introduced specialty-specific variations in acceptance rates, further demonstrating its adaptability across diverse outpatient settings.

However, a decline in acceptance rates was observed during the final months of the study. One potential explanation is the learning effect among physicians. As physicians became more familiar with the system’s diagnostic recommendations and the corresponding medication-diagnosis associations, the occurrence of obvious PIMs likely decreased. Consequently, alerts triggered in the later stages of the study were more likely to involve complex or borderline cases, which physicians were less inclined to accept. Overall, these results demonstrate the success of embedding diagnosis recommendations into alerts, ensuring their relevance, minimizing alert fatigue, and maintaining consistent effectiveness in clinical practice.

### Departmental Influences on Alert Acceptance

Reviewing the variations in acceptance rates across departments, we observed that the complexity of prescribing patterns within each department may be associated with the observed differences in acceptance rates (Table S3 in Multimedia Appendix 4). Ophthalmology achieved the highest rate at 96.59%. A similar study evaluated the artificial intelligence–enhanced CDSS, reported a negative predictive value of 65%-77% for ophthalmology, reflecting its moderate reliability in identifying PIPs [24]. The straightforward nature of diagnoses and prescriptions in ophthalmology likely contributes to the high acceptance rate, as alerts are more actionable and relevant.

In contrast, both rheumatology and surgery exhibited unique patterns in alert outcomes. Rheumatology exhibited the lowest PIP rate (0.18%) and an acceptance rate of 0%. A previous MedGuard study reported an override rate of 85.19% in rheumatology with no actionable outcomes from accepted alerts [22]. Further analysis revealed that high override medication alerts such as omeprazole, often prescribed alongside steroids to prevent gastrointestinal complications, frequently triggered embedded diagnostic recommendations for gastroesophageal reflux disease or peptic ulcers [30]. Similarly, in surgery, alerts for medications such as colchicine, often prescribed for postoperative inflammation, were frequently linked to gout diagnoses, which do not align with the clinical intent [31].

Neurology and psychiatry also demonstrated relatively low acceptance rates. For neurology, the PIP rate was 2.72% aligning with the reported range of 0.42%-2.88% in studies on probabilistic CDSS [24]. While the study reported a negative predictive value for neurology of 40%-60%, our alert acceptance rate was slightly lower at 38.54% [24]. Another study reported a neurology alert acceptance rate of 34.75%, but only 2.13% of accepted alerts led to actual prescription changes [22]. Upon examining the most frequently overridden alerts in neurology, we found that medications such as acetylsalicylic acid and statins often triggered embedded diagnostic recommendations for hyperlipidemia due to missing diagnoses. However, these drugs are primarily used in neurology for cardiovascular prevention, not for treating active disease [32].

In psychiatry, the PIP rate was 0.77% with an alert acceptance rate of 24.74% with issues arising from the diverse dosing ranges of medications such as quetiapine, where dosing variations lead to diverse indications. For example, low doses are used for insomnia or anxiety, while higher doses are prescribed for schizophrenia or bipolar disorder [33,34]. However, the system predominantly associated quetiapine with higher-dose indications, reducing its utility in this specialty.

The infectious diseases department displayed a relatively low acceptance rate (35%), partly due to the stringent requirements of antibiotic stewardship programs (ASPs) [35]. In Taiwan, ASPs require physicians to submit detailed applications for third-line antibiotics and obtain additional approval for fourth-line agents through a separate platform [36]. ASP workflows often overlap with MedGuard alerts, reducing the perceived value of diagnostic recommendations for antibiotics. Physicians typically rely on ASP platforms for pathogen-targeted advice, which may render MedGuard’s suggestions redundant.

### Clinical Implications

We further explored MedGuard’s adaptability by examining four representative medications, including amlodipine, nifedipine, propranolol, and methotrexate, to illustrate its performance in diverse prescribing scenarios (Table 3).

Amlodipine, used exclusively for hypertension, works by blocking calcium channels in the smooth muscle and myocardium, leading to vasodilation and a reduction in blood pressure. In this scenario, MedGuard demonstrated its ability to provide precise embedded diagnostic recommendations in a well-defined clinical context. The system triggered 146 alerts with embedded diagnostic recommendations, achieving an acceptance rate of 63.01%, with all accepted recommendations confirming the diagnosis of essential hypertension (I10).

Nifedipine, a multiuse medication prescribed for hypertension, preterm labor, angina, and Raynaud’s phenomenon, presents a more complex clinical scenario. By relaxing vascular smooth muscle, it promotes vasodilation and reduces blood pressure [37,38]. MedGuard successfully addressed the challenges posed by this multiuse medication, providing specialized embedded diagnostic recommendations tailored to the specific clinical context, whether for cardiology or obstetrics. The system triggered 37 alerts with recommendations, achieving a 56.76% acceptance rate and addressing hypertension (I10) and preterm labor (O60.02), among others. This department-specific approach is essential for medications such as nifedipine, where recommendations need to be aligned with the specialty-specific needs of the clinician.

Propranolol, a nonselective beta-blocker, is used in cardiology, neurology, psychiatry, and other specialties. It is effective for palpitations, anxiety, tremors, cardiac arrhythmias, and migraine prevention. Given its diverse applications, MedGuard triggered 172 alerts with embedded diagnostic recommendations, achieving an acceptance rate of 48.83%. The most accepted recommendation addressed palpitations (R00.2), but the system also provided recommendations for conditions such as cardiac arrhythmia (I49.9), anxiety disorders (F41.9 and F41.1), and migraines (G43.709 and G43.909), demonstrating its ability to provide relevant diagnostic suggestions for a broad range of conditions.

Methotrexate, a high-risk immunomodulator, works by inhibiting dihydrofolate reductase, impairing DNA synthesis and cell division. It is used for autoimmune diseases, chemotherapy, and atopic dermatitis. The system triggered 8 alerts with embedded diagnostic recommendations and with a high acceptance rate of 87.50%. All accepted embedded diagnostic recommendations were for atopic dermatitis (L20.9) showcasing MedGuard’s precision in providing relevant and accurate diagnostic suggestions for high-risk medications.

Collectively, these examples demonstrate MedGuard’s capacity to provide actionable, specialty-relevant recommendations across clinical settings from cardiology and obstetrics to neurology and dermatology. Table S8 in Multimedia Appendix 9 further illustrates how the system supports more informed decision-making by tailoring recommendations to specific workflows.

### Recurring Challenges and Opportunities for Improvement

This study highlights the strengths and limitations of the current CDSS system. A key strength is its effective integration of embedded diagnostic recommendations into medication alerts, which minimizes alert fatigue and enhances diagnostic completeness in clinical practice. Additionally, the enhanced system design outperformed previous MedGuard studies, demonstrating that embedding diagnostic recommendations significantly improves the relevance and utility of alerts for clinical decision-making.

However, improvements are needed in 3 key areas. First, the system should better accommodate preventive medication use, such as omeprazole in rheumatology and colchicine in surgery. Second, it should enhance the ability to link dosage variations to diagnostic recommendations, addressing challenges such as quetiapine’s diverse indications in psychiatry. Finally, better integration with cross-system workflows, such as ASPs in infectious diseases, is essential to minimize redundancies and align with specialty-specific practices. To support broader adoption, establishing standardized application programming interfaces and ensuring interoperability with other CDSS tools could enable seamless integration into clinical workflows. Given MedGuard’s use of standardized data formats, such integration is technically feasible and would facilitate multicenter implementation.

### Limitations

This study demonstrates the successful implementation of a machine learning–based CDSS in a clinical setting, achieving a high acceptance rate for embedded diagnostic recommendations and improving diagnostic completeness. However, it has some limitations. First, the study was conducted in the outpatient departments of a single teaching hospital in Taiwan, which may limit generalizability to other health care systems, regions, or care settings. Second, while the year-long study minimized temporal bias, variations in physician behavior across shifts, differing workloads, or individual factors such as system familiarity and clinical preferences were not specifically analyzed. Additionally, the gradual adoption of MedGuard across participating physicians may have introduced selection bias, as early adopters may differ in prescribing behavior or familiarity with clinical decision support systems compared with those who joined later. Third, although this study demonstrated high acceptance rates and improved diagnostic completeness, it did not evaluate whether these accepted recommendations translated into measurable clinical improvements, such as reduced adverse drug events. Moreover, this study did not include formal qualitative assessments or structured user feedback collection, which could have provided deeper insights into specialty-specific differences in system acceptance. The single-arm design without a control or historical comparator further limits causal inference. Finally, the PIP-level acceptance rate may be overestimated in cases where only a subset of diagnostic recommendations was accepted within a multi-PIM prescription.

### Conclusion

This study highlights the potential of a machine learning–based CDSS to transform outpatient care by improving diagnostic completeness and supporting clinical decision-making. Through actionable and relevant embedded diagnostic recommendations, the system effectively enhanced prescribing practices and minimized alert fatigue, demonstrating its value in optimizing patient safety and health care efficiency. Future efforts should focus on tailoring CDSS systems to specialty-specific prescribing patterns and workflows to further enhance their relevance and acceptance. Future research should explore the impact of embedded diagnostic recommendations on patient outcomes, including multicenter validation, applications in inpatient and emergency settings, and integration with other decision support frameworks such as ASPs. These advancements can further optimize prescription accuracy, reduce medication errors, and support more personalized health care delivery.

## Appendix

Multimedia Appendix 1 TREND checklist.

Multimedia Appendix 2 MedGuard rollout sequence by month of inclusion.

Multimedia Appendix 3 Summary of prescriptions, embedded diagnostic recommendations, and adjustment rates.

Multimedia Appendix 4 Logistic Regression Models Comparing Monthly Differences in PIP and PIM Acceptance.

Multimedia Appendix 5 Departmental Performance in Response to Embedded Diagnostic Recommendations in Alerts.

Multimedia Appendix 6 Logistic Regression Analysis of Department-Specific Differences in PIP Acceptance.

Multimedia Appendix 7 Logistic Regression Analysis of ACT Category-Specific Differences in PIM Acceptance.

Multimedia Appendix 8 Distribution of Embedded Diagnostic Recommendations and Adjusted Diagnoses by ATC Pharmacological Categories.

Multimedia Appendix 9 Examples of Prescription Modifications Before and After Alerts with Embedded Diagnostic Recommendations.

## Abbreviations

ASP: antibiotic stewardship program
ATC: anatomical therapeutic chemical
CDSS: clinical decision support system
CPOE: computerized physician order entry
DM: disease-medication
*ICD-10-CM*: *International Classification of Diseases, Tenth Revision, Clinical Modification*
PIM: potentially inappropriate medication
PIP: potentially inappropriate prescribing
TREND: Transparent Reporting of Evaluations with Nonrandomized Designs

## Data Availability

The datasets generated or analyzed during this study are not publicly available due to institutional restrictions, as the data were obtained from the hospital’s internal prescribing and system backend records, but are available from the corresponding author on reasonable request.
