# Supplementary material for: Evaluation of Diagnostic Recommendations Embedded in Medication Alerts: Prospective Single-Arm Interventional Study
Source: J Med Internet Res. 2025 May 27;27:e70731. doi: 10.2196/70731 (PMC12152430; doi:10.2196/70731)
Supplement: Multimedia Appendix 2 [file jmir_v27i1e70731_app2.docx]

**Table S1 MedGuard rollout sequence by month of inclusion**

| Department | Time of Rollout |
| --- | --- |
| Neurology | January |
| Obstetrics and Gynecology | January |
| Ophthalmology | January |
| Endocrinology | January |
| Cardiology | January |
| Otorhinolaryngology | January |
| Gastroenterology | January |
| Psychiatry | January |
| Rheumatology | January |
| Infectious Disease | January |
| Hematology and Oncology | January |
| Thoracic Surgery | January |
| Neurosurgery | January |
| Pulmonology Medicine | January |
| Family Medicine | March |
| Nephrology | March |
| Surgery | March |
| Dermatology | July |
| Orthopedics | July |
| Others | July |
| Plastic Surgery | July |
| Pediatrics | August |
| Emergency Medicine | September |
