# Supplementary material for: Evaluation of Diagnostic Recommendations Embedded in Medication Alerts: Prospective Single-Arm Interventional Study
Source: J Med Internet Res. 2025 May 27;27:e70731. doi: 10.2196/70731 (PMC12152430; doi:10.2196/70731)
Supplement: Multimedia Appendix 3 [file jmir_v27i1e70731_app3.docx]

**Table S2 Summary of Prescriptions, Embedded Diagnostic Recommendations, and Adjustment Rates**

| Date | Total Prescriptions | Total Medications | PIPs | | | | PIMs | | | |  |
| --- | --- | --- | --- | --- | --- | --- | --- | --- | --- | --- | --- |
|  |  |  | **Recommendations** | | **Acceptances** | | **Recommendations** | | **Acceptances** | |  |
|  | N | N | N | Rate (95% CI) | N | Rate (95% CI) | N | Rate (95% CI) | N | Rate (95% CI) |  |
| 202301 | 9,718 | 27,315 | 198 | 2.04 (2.34, 1.77) | 145 | 73.23 (78.91, 66.67) | 230 | 0.84 (0.96, 0.74) | 146 | 63.48 (69.43, 57.08) |  |
| 202302 | 8,426 | 24,186 | 206 | 2.44 (2.80, 2.14) | 148 | 71.84 (77.54, 65.35) | 245 | 1.01 (1.15, 0.89) | 148 | 60.41 (66.33, 54.17) |  |
| 202303 | 20,332 | 56,699 | 346 | 1.70 (1.89, 1.53) | 245 | 70.81 (75.35, 65.81) | 430 | 0.76 (0.83, 0.69) | 247 | 57.44 (62.03, 52.72) |  |
| 202304 | 30,154 | 83,132 | 450 | 1.49 (1.64, 1.36) | 281 | 62.44 (66.80, 57.88) | 527 | 0.63 (0.69, 0.58) | 282 | 53.51 (57.73, 49.24) |  |
| 202305 | 34,490 | 93,476 | 551 | 1.60 (1.74, 1.47) | 316 | 57.35 (61.41, 53.18) | 636 | 0.68 (0.74, 0.63) | 318 | 50.00 (53.87, 46.13) |  |
| 202306 | 29,788 | 80,475 | 421 | 1.41 (1.55, 1.29) | 256 | 60.81 (65.35, 56.07) | 490 | 0.61 (0.67, 0.56) | 258 | 52.65 (57.04, 48.23) |  |
| 202307 | 37,111 | 105,660 | 860 | 2.32 (2.48, 2.17) | 525 | 61.05 (64.25, 57.75) | 1,027 | 0.97 (1.03, 0.91) | 528 | 51.41 (54.46, 48.36) |  |
| 202308 | 52,770 | 154,748 | 1,591 | 3.01 (3.16, 2.87) | 936 | 58.83 (61.23, 56.39) | 1,966 | 1.27 (1.33, 1.22) | 940 | 47.81 (50.02, 45.61) |  |
| 202309 | 49,403 | 141,637 | 1,433 | 2.90 (3.05, 2.76) | 760 | 53.04 (55.61, 50.45) | 1,847 | 1.30 (1.36, 1.25) | 761 | 41.20 (43.46, 38.98) |  |
| 202310 | 56,250 | 157,398 | 1,415 | 2.52 (2.65, 2.39) | 751 | 53.07 (55.66, 50.47) | 1,725 | 1.10 (1.15, 1.05) | 754 | 43.71 (46.06, 41.39) |  |
| 202311 | 59,145 | 171,238 | 1,290 | 2.18 (2.30, 2.07) | 663 | 51.40 (54.11, 48.67) | 1,591 | 0.93 (0.98, 0.88) | 664 | 41.73 (44.17, 39.33) |  |
| 202312 | 50,971 | 146,717 | 1,245 | 2.44 (2.58, 2.31) | 632 | 50.76 (53.53, 47.99) | 1,523 | 1.04 (1.09, 0.99) | 635 | 41.69 (44.19, 39.24) |  |
| **Sum** | **438,558** | **1,242,681** | **10,006** | **2.28 (2.33, 2.24)** | **5,658** | **56.55 (57.51, 55.57)** | **12,237** | **0.98 (1.00, 0.97)** | **5,681** | **46.42 (47.54, 45.54)** |  |
| PIPs: potentially inappropriate prescriptions; PIMs: potentially inappropriate medications | | | | | | | | | | | |
