# Supplementary material for: Evaluation of Diagnostic Recommendations Embedded in Medication Alerts: Prospective Single-Arm Interventional Study
Source: J Med Internet Res. 2025 May 27;27:e70731. doi: 10.2196/70731 (PMC12152430; doi:10.2196/70731)
Supplement: Multimedia Appendix 4 [file jmir_v27i1e70731_app4.docx]

**Table S3. Logistic Regression Models Comparing Monthly Differences in PIP and PIM Acceptance**

|  | Model 1: PIP Acceptance | | Model 2 PIM Acceptance | |
| --- | --- | --- | --- | --- |
| Variable | Odds ratio (95% CI) | p-value | Odds ratio (95% CI) | p-value |
| Age | 0.986 (0.984, 0.988) | <.0001 | 0.988 (0.987, 0.990) | <.0001 |
| Sex | 0.537 (0.491, 0.586) | <.0001 | 0.669 (0.617, 0.725) | <.0001 |
| Time (ref: Dec) |  |  |  |  |
| Jan | 2.785 (1.983, 3.912) | <.0001 | 2.551 (1.908, 3.411) | <.0001 |
| Feb | 2.503 (1.804, 3.474) | <.0001 | 2.195 (1.662, 2.901) | <.0001 |
| Mar | 2.355 (1.812, 3.061) | <.0001 | 1.900 (1.526, 2.366) | <.0001 |
| Apr | 1.795 (1.432, 2.251) | <.0001 | 1.773 (1.449, 2.171) | <.0001 |
| May | 1.469 (1.194, 1.807) | .0003 | 1.534 (1.270, 1.852) | <.0001 |
| Jun | 1.639 (1.302, 2.064) | <.0001 | 1.681 (1.366, 2.068) | <.0001 |
| Jul | 1.591 (1.329, 1.906) | <.0001 | 1.527 (1.300, 1.795) | <.0001 |
| Aug | 1.438 (1.234, 1.676) | <.0001 | 1.316 (1.148, 1.509) | <.0001 |
| Sep | 1.125 (0.963, 1.314) | .1386 | 1.004 (0.873, 1.154) | .9597 |
| Oct | 1.114 (0.953, 1.302) | .1752 | 1.101 (0.956, 1.268) | .1829 |
| Nov | 1.013 (0.864, 1.188) | .8765 | 0.992 (0.859, 1.146) | .9141 |
