# Supplementary material for: Evaluation of Diagnostic Recommendations Embedded in Medication Alerts: Prospective Single-Arm Interventional Study
Source: J Med Internet Res. 2025 May 27;27:e70731. doi: 10.2196/70731 (PMC12152430; doi:10.2196/70731)
Supplement: Multimedia Appendix 5 [file jmir_v27i1e70731_app5.docx]

**Table S4. Departmental Performance in Response to Embedded Diagnostic Recommendations in Alerts**

| Department | Total Prescriptions | Embedded Diagnostic Recommendations | | Acceptances | |
| --- | --- | --- | --- | --- | --- |
|  | N | N | Rate (95% CI) | N (%) | Rate (95%CI) |
| Neurology | 100,207 | 2,727 | 2.72 (2.82, 2.62) | 1,051 | 38.54 (40.38, 36.73) |
| Obstetrics and Gynecology | 60,975 | 2,252 | 3.69 (3.85, 3.55) | 2,027 | 90.01 (91.18, 88.70) |
| Ophthalmology | 49,608 | 352 | 0.71 (0.79, 0.64) | 340 | 96.59 (98.04, 94.14) |
| Endocrinology | 44,977 | 577 | 1.28 (1.39, 1.18) | 366 | 63.43 (67.26, 59.42) |
| Cardiology | 35,388 | 847 | 2.39 (2.56, 2.24) | 427 | 50.41 (53.77, 47.05) |
| Otorhinolaryngology | 34,541 | 1,314 | 3.80 (4.01, 3.61) | 760 | 57.84 (60.48, 55.15) |
| Gastroenterology | 27,999 | 306 | 1.09 (1.22, 0.98) | 206 | 67.32 (72.33, 61.88) |
| Psychiatry | 24,608 | 190 | 0.77 (0.89, 0.67) | 47 | 24.74 (31.33, 19.14) |
| Dermatology | 10,222 | 23 | 0.23 (0.34, 0.15) | 14 | 60.87 (77.84, 40.79) |
| Rheumatology | 8,454 | 15 | 0.18 (0.29, 0.11) | 0 | 0.00 (20.39, 0.00) |
| Infectious Disease | 8,246 | 400 | 4.85 (5.34, 4.41) | 140 | 35.00 (39.80, 30.49) |
| Hematology and Oncology | 7,924 | 384 | 4.85 (5.34, 4.39) | 42 | 10.94 (14.45, 8.19) |
| Pediatrics | 7,610 | 105 | 1.38 (1.67, 1.14) | 86 | 81.90 (88.10, 73.46) |
| Thoracic Surgery | 7,152 | 155 | 2.17 (2.53, 1.85) | 69 | 44.52 (52.38, 36.92) |
| Orthopedics | 4,998 | 217 | 4.34 (4.94, 3.81) | 9 | 4.15 (7.69, 2.20) |
| Family Medicine | 2,038 | 26 | 1.28 (1.86, 0.87) | 23 | 88.46 (96.00, 71.02) |
| Others | 1,941 | 50 | 2.58 (3.38, 1.96) | 45 | 90.00 (95.65, 78.64) |
| Neurosurgery | 578 | 33 | 5.71 (7.91, 4.09) | 2 | 6.06 (19.61, 1.68) |
| Nephrology | 500 | 1 | 0.20 (1.12, 0.04) | 0 | 0.00 (79.35, 0.00) |
| Surgery | 250 | 27 | 10.80 (15.26, 7.53) | 0 | 0.00 (12.46, 0.00) |
| Pulmonology Medicine | 198 | 4 | 2.02 (5.08, 0.79) | 4 | 100.00 (100.00, 51.01) |
| Plastic Surgery | 74 | 0 | 0.00 (4.94, 0.00) | N/A | N/A |
| Emergency Medicine | 70 | 1 | 1.43 (7.66, 0.25) | 0 | 0.00 (79.35, 0.00) |
| Total | 438,558 | 10,006 | 2.28 (2.33, 2.24) | 5,658 | 56.55 (57.51, 55.57) |

Note: N/A: Not applicable
