# Supplementary material for: Evaluation of Diagnostic Recommendations Embedded in Medication Alerts: Prospective Single-Arm Interventional Study
Source: J Med Internet Res. 2025 May 27;27:e70731. doi: 10.2196/70731 (PMC12152430; doi:10.2196/70731)
Supplement: Multimedia Appendix 6 [file jmir_v27i1e70731_app6.docx]

**Table S5. Logistic Regression Analysis of Department-Specific Differences in PIP Acceptance**

|  | **PIP Acceptance** | |
| --- | --- | --- |
| **Variable** | **Odds ratio (95% CI)** | **p-value** |
| Age | 1.004 (1.002, 1.007) | .0018 |
| Sex | 0.900 (0.815, 0.994) | .0384 |
| Department (ref: Neurology) |  |  |
| Cardiology | 1.629 (1.394, 1.903) | <.0001 |
| Dermatology | 2.907 (1.245, 6.785) | .0136 |
| Otorhinolaryngology | 2.411 (2.079, 2.797) | <.0001 |
| Emergency Medicine | <0.001 (<0.001, >999.999) | .9911 |
| Endocrinology | 2.851 (2.365, 3.437) | <.0001 |
| Family Medicine | 12.943 (3.873, 43.254) | <.0001 |
| Gastroenterology | 3.430 (2.666, 4.414) | <.0001 |
| General Surgery | <0.001 (<0.001, >999.999) | .9541 |
| Hematology and Oncology | 0.197 (0.142, 0.274) | <.0001 |
| Infectious Disease | 0.923 (0.739, 1.153) | .4815 |
| Nephrology | <0.001 (<0.001, >999.999) | .9911 |
| Neurosurgery | 0.110 (0.026, 0.463) | .0026 |
| Obstetrics and Gynecology | 15.222 (12.769, 18.147) | <.0001 |
| Ophthalmology | 47.502 (26.523, 85.074) | <.0001 |
| Orthopedics | 0.071 (0.036, 0.139) | <.0001 |
| Pediatrics | 9.274 (5.457, 15.761) | <.0001 |
| Psychiatry | 0.560 (0.398, 0.789) | .0009 |
| Pulmonology Medicine | >999.999 (<0.001, >999) | .9808 |
| Rheumatology | <0.001 (<0.001, >999.999) | .9656 |
| Thoracic Surgery | 1.321 (0.953, 1.831) | .0950 |
| Others | 15.319 (6.050, 38.787) | <.0001 |

Note: Plastic Surgery was excluded from the regression model because no PIP recommendations were triggered in this department, making acceptance analysis not applicable.
