# Supplementary material for: Evaluation of Diagnostic Recommendations Embedded in Medication Alerts: Prospective Single-Arm Interventional Study
Source: J Med Internet Res. 2025 May 27;27:e70731. doi: 10.2196/70731 (PMC12152430; doi:10.2196/70731)
Supplement: Multimedia Appendix 7 [file jmir_v27i1e70731_app7.docx]

**Table S6. Logistic Regression Analysis of ACT Category-Specific Differences in PIM Acceptance**

|  | PIM Acceptance | |
| --- | --- | --- |
| Variable | Odds ratio (95% CI) | p-value |
| Age | 0.990 (0.988, 0.992) | <.0001 |
| Sex | 0.731 (0.672, 0.795) | <.0001 |
| ACT code category (ref: N) |  |  |
| A | 2.678 (2.321, 3.089) | <.0001 |
| B | 1.547 (1.359, 1.760) | <.0001 |
| C | 3.050 (2.680, 3.472) | <.0001 |
| G | 7.461 (6.122, 9.093) | <.0001 |
| H | 0.552 (0.451, 0.674) | <.0001 |
| J | 2.861 (2.476, 3.305) | <.0001 |
| L | 1.355 (0.829, 2.215) | .2260 |
| M | 2.018 (1.716, 2.374) | <.0001 |
| P | 2.396 (1.350, 4.251) | .0028 |
| R | 2.352 (1.990, 2.780) | <.0001 |
| S | 6.703 (5.253, 8.552) | <.0001 |
