# Supplementary material for: Evaluation of Diagnostic Recommendations Embedded in Medication Alerts: Prospective Single-Arm Interventional Study
Source: J Med Internet Res. 2025 May 27;27:e70731. doi: 10.2196/70731 (PMC12152430; doi:10.2196/70731)
Supplement: Multimedia Appendix 8 [file jmir_v27i1e70731_app8.docx]

**Table S7. Distribution of Embedded Diagnostic Recommendations and Adjusted Diagnoses by ATC Pharmacological Categories**

| ATC  group | Pharmacological Categories | Embedded Diagnostic Recommendations | Acceptances | |
| --- | --- | --- | --- | --- |
|  |  | N (%) | N | Rate |
| N | Nervous system | 2,591 (21.17%) | 717 | 27.67% |
| C | Cardiovascular system | 1,795 (14.67%) | 943 | 52.53% |
| B | Blood and blood forming organs | 1,794 (14.66%) | 713 | 39.74% |
| A | Alimentary tract and metabolism | 1,262 (10.31%) | 653 | 51.74% |
| J | Anti-infectives for systemic use | 1,189 (9.72%) | 745 | 62.66% |
| M | Musculoskeletal system | 819 (6.69%) | 390 | 47.62% |
| R | Respiratory system | 802 (6.55%) | 413 | 51.50% |
| G | Genito-urinary system and sex hormone | 766 (6.26%) | 605 | 78.98% |
| H | System hormonal preparations, excluding sex hormones and insulins | 684 (5.59%) | 153 | 22.37% |
| S | Sensory organs | 422 (3.45%) | 295 | 69.91% |
| L | Immunomodulating agents | 62 (0.51%) | 26 | 41.94% |
