# Supplementary material for: Evaluation of Diagnostic Recommendations Embedded in Medication Alerts: Prospective Single-Arm Interventional Study
Source: J Med Internet Res. 2025 May 27;27:e70731. doi: 10.2196/70731 (PMC12152430; doi:10.2196/70731)
Supplement: Multimedia Appendix 9 [file jmir_v27i1e70731_app9.docx]

**Table S8 Examples of Prescription Modifications Before and After Alerts with Embedded Diagnostic Recommendations**

| **Department** | **PIP Alerts by MedGuard** | **Modified Prescriptions After Alerts** |
| --- | --- | --- |
| **Neurology** | **Diagnosis (ICD-10)**   - Dizziness and giddiness (R42) - Insomnia, unspecified (G47.00)   **Medications (ATC code)**   - Amlodipine (C08CA01) - Diphenidol (N07CA91) - Brotizolam (N05CD09) | **Diagnosis (ICD-10)**   - Dizziness and giddiness (R42) - Insomnia, unspecified (G47.00) - Essential (primary) hypertension (I10)   **Medications (ATC code)**   - Amlodipine (C08CA01) - Diphenidol (N07CA91) - Brotizolam (N05CD09) |
| **Obstetrics and Gynecology** | **Diagnosis (ICD-10)**   - Maternal care for breech presentation, not applicable or unspecified (O32.1XX0) - Anemia, unspecified (D64.9)   **Medications (ATC code)**   - Iron and multivitamins (B03AE03) - Nifedipine (C08CA05) | **Diagnosis (ICD-10)**   - Maternal care for breech presentation, not applicable or unspecified (O32.1XX0) - Anemia, unspecified (D64.9) - Preterm labor without delivery, second trimester (O60.02)   **Medications (ATC code)**   - Iron and multivitamins (B03AE03) - Nifedipine (C08CA05) |
| **Endocrinology** | **Diagnosis (ICD-10)**   - Idiopathic hypoparathyroidism (E20.0)   **Medications (ATC code)**   - Calcitriol (A11CC04) - Levothyroxine (H03AA01) - Propranolol (C07AA05) | **Diagnosis (ICD-10)**   - Idiopathic hypoparathyroidism (E20.0) - Palpitations (R00.2)   **Medications (ATC code)**   - Calcitriol (A11CC04) - Levothyroxine (H03AA01) - Propranolol (C07AA05) |
| **Dermatology** | **Diagnosis (ICD-10)**   - Contact dermatitis, unspecified cause (L25.9)   **Medications (ATC code)**   - Dexchlorpheniramine (R06AB02) - Methotrexate (L01BA01) - Folic acid (B03BB01) | **Diagnosis (ICD-10)**   - Contact dermatitis, unspecified cause (L25.9) - Atopic dermatitis, unspecified (L20.9)   **Medications (ATC code)**   - Dexchlorpheniramine (R06AB02) - Methotrexate (L01BA01) - Folic acid (B03BB01) |

**Note:** Red text highlights the initial alerts generated by the system, while blue text indicates the recommendations accepted and implemented by physicians in response to these alerts.
